# Ganoderic Acid A Prevented Osteoporosis by Modulating the PIK3CA/p‐Akt/TWIST1 Signaling Pathway

**DOI:** 10.1002/fsn3.70177

**Published:** 2025-04-14

**Authors:** Jianyu Zhao, Ying Fan, Hao Li, Changyuan Wang, Sihang Fan, Huijun Sun, Mozhen Liu

**Affiliations:** ^1^ Department of Orthopaedics, the First Affiliated Hospital Dalian Medical University Dalian China; ^2^ Department of Clinical Pharmacology, College of Pharmacy Dalian Medical University Dalian China

**Keywords:** ganoderic acid a, network pharmacology, osteoporosis, p‐Akt, PIK3CA, TWIST1

## Abstract

Osteoporosis is a disorder of decreased bone mass, microarchitectural deterioration, and fragility fractures. Ganoderma lucidum has been reported to have a variety of pharmacological activities, including immune regulation, anti‐inflammation, antioxidation, sedative hypnosis, blood sugar and lipid regulation, and so on. However, the effective ingredients and the underlying mechanism of Ganoderma lucidum against osteoporosis are rarely clarified. Ganoderic acid A (GA‐A), a triterpenoid, is one of the main components of Ganoderma lucidum. Our previous preliminary bioinformatic study found that it may affect bone metabolism, and it has been reported that GA‐A has anti‐osteoporosis potential via regulating MC3T3‐E1 cells' osteogenic differentiation activity. Therefore, the aim of this study is to investigate the effects of Ganoderic acid A in preventing osteoporosis and uncover the potential mechanisms. In vivo, the 8‐week‐old C57BL/6J female mice were used to establish the osteoporosis model by ovariectomy (OVX). Two cell lines, MC3T3‐E1 cells and primary osteoblasts, were used and induced with hydrogen peroxide (H_2_O_2_) to the state of oxidative stress in osteoporosis in vitro. We showed that Ganoderic acid A could inhibit OVX‐induced bone loss in a dose‐dependent manner and promote H_2_O_2_‐induced osteogenic differentiation of primary osteoblasts and MC3T3‐E1 cells. The mechanism‐related signaling pathways were identified by network pharmacology screening and verified by bioinformatics. Results predicted that the target of Ganoderic acid A might be PIK3CA. Mechanistically, we found that PIK3CA activated the Akt receptor, then inhibited the expression of TWIST1 in the osteoblasts to up‐regulate the protein expression of the osteogenic‐related markers. Our results suggested that Ganoderic acid A could prevent OVX‐induced osteoporosis and promote H_2_O_2_‐induced osteogenic differentiation of primary osteoblasts and MC3T3‐E1 cells. Ganoderic acid A might play an important role in the prevention of osteoporosis by modulating the PIK3CA/p‐Akt/TWIST1 signaling pathway.

## Graphical abstract

Ganoderic acid A can prevent osteoporosis. Ganoderic acid A alleviates oxidative stress in vivo in ovariectomy‐induced mice. Ganoderic acid A promotes H_2_O_2_‐induced osteogenic differentiation of primary osteoblasts and MC3T3‐E1 cells.

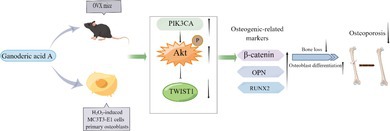

**Abbreviations**
ALP: alkaline phosphatase
ARS: alizarin red staining
OPN: osteopontin
p‐Akt: v‐akt murine thymoma viral oncogene homolog 1
PIK3CA: PI3 kinase p110 subunit alpha
RUNX2: runt‐related transcription factor 2
TWIST1: twist homolog 1

## 1 Introduction

Osteoporosis (OP) is an age‐related chronic disease with reduced mineralized bone density and an increased risk of fracture (Föger‐Samwald et al. 2022). The occurrence and development of osteoporosis are closely related to the imbalance between bone formation by osteoblasts and bone loss by osteoclasts (Ferbebouh et al. 2021). Although osteoporosis has become a major public health problem worldwide, less desirable drugs are available clinically (Tao et al. 2024); (Song et al. 2022).

The existing therapeutic drugs for OP clinically include bone supplements (Calcium, vitamin D, etc.) (Reid and Bolland 2019), anti‐osteoclast drugs (such as alendronate sodium, zoledronic acid, salinic acid, etc.) (Lorentzon 2019), anti‐resorption drugs (such as salmon calcitonin) (Henriksen et al. 2010) and estrogen receptor modulators (such as raloxifene) (Clemett and Spencer 2000), etc. However, long‐term use of these drugs can result in unneglectable side effects, for example, nausea, vomiting, nervous system side effects such as headache, and in severe cases may even lead to malignant tumors and other diseases (Modi et al. 2015; Gambrell Jr. 1986). Therefore, new therapeutic drugs or programs are still needed to reduce or eliminate the pain suffered by patients due to osteoporosis.

Traditional Chinese medicine (TCM) is a treasure of Chinese civilization, and there are rich treatment methods for the occurrence and development of osteoporosis (Lin et al. 2017). From the perspective of traditional Chinese medicine, osteoporosis belongs to the category of diseases named such as “bone weakness” and “fracture” and its fundamental reason is the imbalance of Yin and Yang (Shen et al. 2020; Yang et al. 2011), which is characterized by the overall state of Yang deficiency and Yin excess (Chen et al. 1999). Nowadays, there are more and more studies on the treatment of osteoporosis with herbal medicine. Compared with Western medicine, which has many more side effects, the dialectical treatment of traditional Chinese medicine is easier for patients to recover (Herrmann et al. 2020; Zhang 2015).

As a traditional Chinese medicine, Ganoderma lucidum is known as the “king of traditional Chinese medicine” (Seweryn et al. 2021). It can strengthen Yang, relieve tendons, calm the mind, benefit qi, and can also produce anti‐osteoporosis effects (Ahmad 2018). As for the anti‐osteoporosis effects of Ganoderma lucidum, it has been reported that it can produce therapeutic effects by inhibiting MAPKs (Tran et al. 2019) and NFATc1, and weakening RANKL‐mediated osteoclast generation (Yang and Yang 2019). Ganoderic acid A(GA‐A)is a triterpenoid substance, which is one of the main active ingredients of Ganoderma lucidum (Cao et al. 2017). Therefore, it is reasonable to assume that GA‐A is mainly responsible for the anti‐osteoporosis effects of Ganoderma lucidum. Therefore, we hypothesized that GA‐A is the main component of Ganoderma lucidum in combating osteoporosis to increase bone mass by promoting osteogenic differentiation.

In order to verify this conjecture, we used bioinformatics to screen the targets of Ganoderic acid A on osteoporosis and identified PIK3CA as the main target of GA‐A against osteoporosis. PIK3CA is a PI3K/Akt signaling pathway‐related protein, which can encode p110α protein, catalyze PI3K signal transduction, and activate Akt to the phosphorylated form (Asati et al. 2016). P‐Akt in the activated state can inhibit the expression of its downstream TWIST1 protein (Liu and Gao 2019). A large number of literary studies have shown that PI3K/Akt, as a classical signaling pathway, can affect the pathological changes of many diseases, including the inhibition of OP (Cheng et al. 2017; Xu et al. 2020; Ma et al. 2021). In addition, the TWIST1 protein has also been reported in many literatures to inhibit bone formation in bone‐related diseases (Li et al. 2023). Therefore, it is reasonable to speculate that Ganoderic acid A can promote bone formation in osteoblasts by activating PIK3CA to catalyze the PI3K/Akt signaling pathway and then inhibiting the protein expression of TWIST1, thus playing a positive role in preventing and treating osteoporosis.

## 2 Materials and Methods

### 2.1 Reagents

Ganoderic acid A (batch number 20180327, and purity [HPLC 256 nm] > 99.9%) was purchased from Baoji Chenguang Biotechnology Co., LTD. Dimethyl sulfoxide (DMSO) was purchased from Sigma. Tween80 and Tween20 were purchased from Solarbio Biotech Co., LTD (Beijing, China). LY294002 (purity [HPLC] > 99.92%) from MedChemExpress(MCE), and MK2206 (purity [HPLC] > 99.59%) were purchased from Shanghai Taoshu Biotechnology Co., LTD. Serum detection kits (LDH, MDA, SOD, AKP) were purchased from Nanjing Jiancheng Biotechnology Co., LTD. Antibodies against OPN, RUNX2, β‐catenin, TWIST1, AKT, and p‐Akt (ser473) were purchased from PROTEINTECH GROUP, INC., and the antibody against PIK3CA was purchased from Beijing Bioss Biotechnology Co., LTD. Horseradish enzyme‐labeled goat anti‐rabbit and anti‐mouse IgG secondary antibodies were purchased from PROTEINTECH GROUP, INC. DMEM medium was purchased from Seven Innovative Biotechnology Co., LTD., and fetal bovine serum was purchased from Shanghai Yamei Biotechnology Co., LTD.

### 2.2 Bioinformatics Analysis

The GA‐A‐related targets were obtained through BATMAN‐TCM (Score cut off ≥ 20, http://bionet.ncpsb.org/batman‐tcm/), Super Pred (http://prediction.charite.de/), and Swiss Target Prediction (*p* ≤ 0.5, http://www.swisstargetprediction.ch/) websites; The OP related targets were obtained through the Gene—Cards (score ≥ 1, https://www.genecards.org/), Online Mendelian Inheritance in Man (OMIM, https://www.omim.org/), and DisGe—NET (score ≥ 0.4, https://www.disgenet.org/) web sites. The Venn diagram tool (https://bioinfogp.cnb.csic.es/tools/venny/index.html) was used to obtain the GA/OP‐related targets. The obtained drug disease target proteins were imported into the online database STRING (https://string‐db.org/) to construct the PPI (protein–protein interaction) network, and Cytoscape software (version 3.7.2) was used to further visualize the PPI network to find the central targets.

### 2.3 Molecular Docking

The structure of the target protein (PIK3CA) was extracted from the RCSB bank database (PDB, http://www.rcsb.org/pdb/home/home.do. PDB ID: 7R9V), which is a database of the three‐dimensional structures of biomacromolecules (proteins, nucleic acids, sugars). The 3D chemical structure of GA‐A was obtained from the ZINC database (http://zinc.docking.org/). Before molecular docking, the monomeric protein structure PIK3CA was picked out from the original PDB file and carried out energy minimization and water removal, and hydrogenation operations. Molecular docking of proteins to GA‐A was performed using Autodock software (Version 4.2.1), and the free binding energy and binding status were determined. The docking pocket refers to the small ligand molecules already presented in the PDB file.

### 2.4 Detection of Serum Indicators

The levels of the serum indicators including malondialdehyde (MDA), alkaline phosphatase (ALP), lactate dehydrogenase (LDH), and superoxide dismutase (SOD) were measured by detection kits based on the manufacturer's instructions (Nanjing Jiancheng Bioengineering Ltd., Nanjing, China).

### 2.5 Cell Culture

In this study, primary osteoblasts (POB) and MC3T3‐E1 (MC) cells were used. POB were isolated from the cranium of 3–7‐day‐old mice and digested with type‐II collagenase and then transferred to cell flasks for culture. After 1–3 days, the cells were reseeded, and the medium was replaced every other day. MC cells were purchased from the Shanghai Institute of Cell Biology, Chinese Academy of Sciences. Both cell lines were cultured in a DMEM medium containing 10% FBS and 100 U/mL penicillin–streptomycin. Cell culture flasks were incubated in incubators at 37°C with 5% CO_2_.

### 2.6 Animal Experiments

Eight‐week‐old C57BL/6J female mice (purchased from Liaoning Changsheng Biotechnology Co., LTD.) were selected. After 1 week of adaptive feeding, they were randomly divided into 5 groups, with 10 mice in each group, which were the control group, model group, low (5 mg/kg), medium (10 mg/kg), and high (20 mg/kg) doses of GA‐A groups. The doses for the mice experiments were determined by referring to previous studies based on mice models (Lu et al. 2021; Abudumijiti et al. 2021). The animals were reared in a house with constant temperature and were provided with food and water ad libitum during the study. Ovariectomy (OVX) was used to establish the postmenopausal osteoporosis model, and the control group underwent sham surgery, from which only part of the fat tissue around the ovaries was removed. One week after the operation, the mice in the low, medium, and high doses of GA‐A groups were treated with different doses of GA‐A by intraperitoneal injection. Eight weeks later, the mice were sacrificed under an over‐dose of anesthesia (Urethane 1.2 g/kg), and the serum and bone tissues were collected and stored in a refrigerator at −80°C for further analysis. All animal experiments were conducted according to the Guide for the Care and Use of Laboratory Animals and approved by the Animal Research Ethics Committee of Dalian Medical University (Dalian, China; ethical permit No. CSE202303003). All efforts were made to minimize the number and suffering of the animals used.

### 2.7 Cell Proliferation Assay

Both kinds of cells were seeded in 96‐well plates. When the cell density was about 80% confluence (8 × 10^3 cells per well), different doses of Ganoderic acid A (0, 5, 10, 20, 40, 80, 160 μM, dissolved in DMSO) were given for 24 h, and then Cell Counting Kit‐8 (cck8) reagent (purchased from Beyotime Biotechnology, Shanghai, China) was given for treatment. After 4 h, the drug toxicity of Ganoderic acid A was detected by measuring the absorbance (a) at 450 nm using a thermo354 microplate reader (Thermo Fisher Scientific, China). Hydrogen peroxide (H_2_O_2_) at a certain concentration can cause oxidative stress damage to cells; thus, it was used for the in vitro model. Different concentrations of hydrogen peroxide (0, 100, 150, 200, 250, 300 μM, dissolved in DMEM) were added for 4 h, and the cck8 reagent was added after 4–6 h to detect the effect of hydrogen peroxide on cell survival rate.

### 2.8 Western Blot

The cell or tissue proteins were mixed with 2 × loading buffer and then separated by electrophoresis. The corresponding gel blocks were cut off according to the different molecular weights labeled by the markers, and the proteins were transferred to the PVDF membranes by membrane transfer apparatus. After completion of the transfer, the membranes were blocked with 5% skim milk powder for 2 h, then cleaned with TTBS and incubated with the primary antibodies against the specific targets in a refrigerator at 4°C overnight. The following day, the secondary antibodies conjugated with horseradish peroxidase were incubated for 1.5 h in a water bath at 37°C. After the incubation was completed, the membranes were rinsed with TTBS and, using a gel imager (BioSpevtrum, USA), the protein expressions on PVDF membranes were observed.

### 2.9 Immunofluorescence

The 12‐well plates of pretreated cells were removed, the culture medium was discarded, rinsed with PBS, and the cells were fixed with 4% paraformaldehyde for 30 min. Then the cells were treated with 0.1% TritonX‐100 for 20 min and blocked with 1% BSA for 30 min. After washing, the cells were incubated with the primary antibodies overnight. On the following day, the primary antibody was washed off and incubated with a fluorescent secondary antibody in the dark for 1 h at 37°C in a water bath, followed by 10 min with DAPI in the dark. The images were finally captured by using a fluorescence inverted microscope (Olympus, Japan).

### 2.10 Osteogenic Induction

MC cells were used for osteogenic induction, and the osteogenic induction solution consisted of dexamethasone sodium phosphate injection (5 μL, 1 mL:5 mg), β‐glycerophosphate sodium (1.5 g), and vitamin C (4.3 mg), which were added to DMEM blank medium for later use. Cells were seeded in 12‐well plates and treated with ganoderic acid A for another 24 h after the addition of hydrogen peroxide for 4–6 h, and then replaced with osteogenic induction solution diluted 100‐fold as described above and replaced every other day. To detect osteogenic differentiation activity, an alkaline phosphatase staining was performed after 14 days of induction, and an alizarin red staining was performed after 21 days of induction to detect the mineralized precipitation of the cells; the specific experimental operations were referred to the kit instructions.

### 2.11 Gene Silencing

MC cells were seeded in six‐well plates at a density of 1 × 10^6^ per well, and the designed si‐RNA sequence (sense 5′‐3′:GGUACAUCGACUUCCUGUATT and antisense 5′‐3′:UACAGGAAGUCGAUGUACCTT for Twist1 silencing, sense 5′‐3′:GCAGAGCAAUGUAUGUCUATT and antisense 5′‐3′:UAGACAUACAUUGCUCUGCTT for PIK3CA silencing; purchased from Jima Gene Co., LTD., Suzhou, China) was transfected into the cells via lipo2000 according to the manufacturer's recommended conditions. After 4–6 h of transfection, the transfection medium was removed and the cells were re‐cultured in a complete medium for24 h. Then the proteins were extracted for Western blot assay to detect the silencing of Twist1 and the expression changes of related proteins.

### 2.12 Dose–Response Cellular Thermal Shift Assay (CETSA)

Cellular Thermal shift assays (CETSA) can be used to understand the thermal stability of proteins upon binding to ligands. Briefly, the cells were heated to 65°C for 5 min after being treated with different concentrations of GA‐A or solvent control, then the thermal stability of target proteins was detected via WB assay.

### 2.13 Statistical Analysis

GraphPad Prism 8.0 software was used for the statistical analysis of experimental data, and the results obtained by the analysis were expressed as mean ± standard deviation (SD). The student t‐test was used for comparisons between any two different groups, one‐way analysis of variance was used for more than two groups, and the resulting P value < 0.05 was considered statistically significant.

## 3 Results

### 3.1 GA‐A Dose‐Dependently Reverses the Bone Loss and Changes in Bone Microstructure in the OVX Mice

**FIGURE 1:**
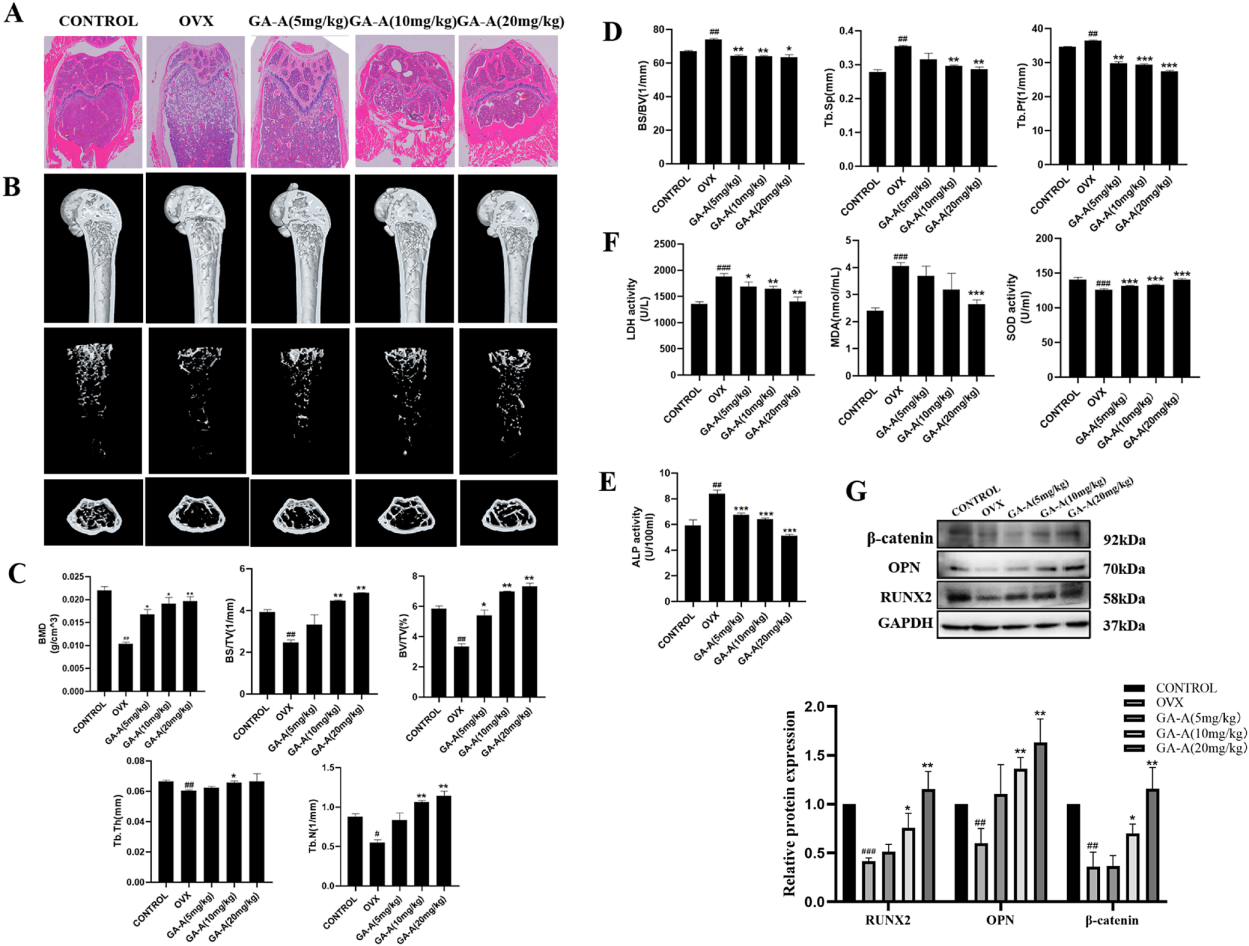
GA‐A inhibited bone loss in OVX mice. (A) Representative images of H&E staining. (B) Representative 3D micro‐CT analysis images of the femur were reconstructed. (C) The BMD, BV/TV, BS/TV, Tb. Th, Tb. N values of micro‐CT analysis were evaluated. (D) BS/BV, Tb. Sp, and Tb. Pf values of micro‐CT analysis were evaluated. (E) Effect of GA‐A on serum ALP activity of mice. (F) GA‐A inhibited OVX‐induced increase in LDH and SOD; and inhibited OVX‐induced decrease in MDA. (G) Protein expression of osteogenesis‐related markers RUNX2, OPN, and β‐catenin in mice. *n* = 6, #*p* < 0.05, ##*p* < 0.01 and ###*p* < 0.001 compared with the control group. *p < 0.05, **p < 0.01 and ***p < 0.001 compared with the OVX group.

To investigate the effects of Ganoderic acid A on osteoporosis, the femurs of the mice were selected for H&E staining, and the results showed that the bone tissue in the OVX group showed increased fat cavities, discontinuous bone trabeculae, and fracture of bone microstructure compared with the sham‐operated group, indicating the bone loss and decreased osteogenesis in the OVX mice, and the model of osteoporosis was successfully established. In contrast, the internal cavities of the femur and the fracture of the bone microstructure were obviously improved in the Ganoderic acid A treatment groups (Figure 1A). The results of the micro‐CT examination of the femur showed that the bone mineral density of the femur in the OVX mice was significantly reduced and the bone microstructure was severely damaged (Figure 1B). Among the specific bone microstructural measurements, bone mineral density (BMD), bone volume fraction (BV/TV), bone surface area and tissue volume ratio (BS/TV), trabecular thickness (Tb.Th), and trabecular number (Tb.N) were all found to be markedly decreased in the OVX group, which could be reversed by GA‐A treatment (Figure 1C). In contrast, parameters including the trabecular surface area to volume ratio (BS/BV), trabecular separation (Tb.Sp), and trabecular pattern factor (Tb.Pf) were increased obviously in the OVX group compared with the sham group and were decreased significantly after GA‐A treatment (Figure 1D).

The activity of ALP detecting results showed that compared with the sham group, the activity of ALP in the OVX group was significantly increased, indicating the increased bone loss (Llorente‐Pelayo et al. 2020). However, the activity of ALP was gradually decreased after GA‐A intervention (Figure 1E). In addition, in order to verify the oxidative stress damage caused by OVX and the antagonizing effects of GA‐A, the oxidative stress‐related kits were adopted to examine the oxidative stress‐related markers of LDH, MDA, SOD levels. The results showed that the activities of LDH and MDA in the OVX group were significantly higher, while the activity of SOD was markedly lower than those in the sham group. However, after the administration of GA‐A, the above oxidative stress‐related markers were significantly reversed dose dependently, indicating the anti‐oxidative effects of GA‐A in preventing against OVX‐induced osteoporosis (Figure 1F).

Western blotting analysis of the protein expression of the osteogenic‐related markers RUNX2, OPN, and β‐catenin in the mice femur showed that the levels of all the osteogenic markers were decreased in the OVX group, but they were significantly increased after GA‐A administration (Figure 1G). Collectively, the above results indicated that GA‐A could combat bone loss and changes in the bone microstructure in the OVX mice.

### 3.2 Network Pharmacology and Molecular Docking Analysis

**FIGURE 2:**
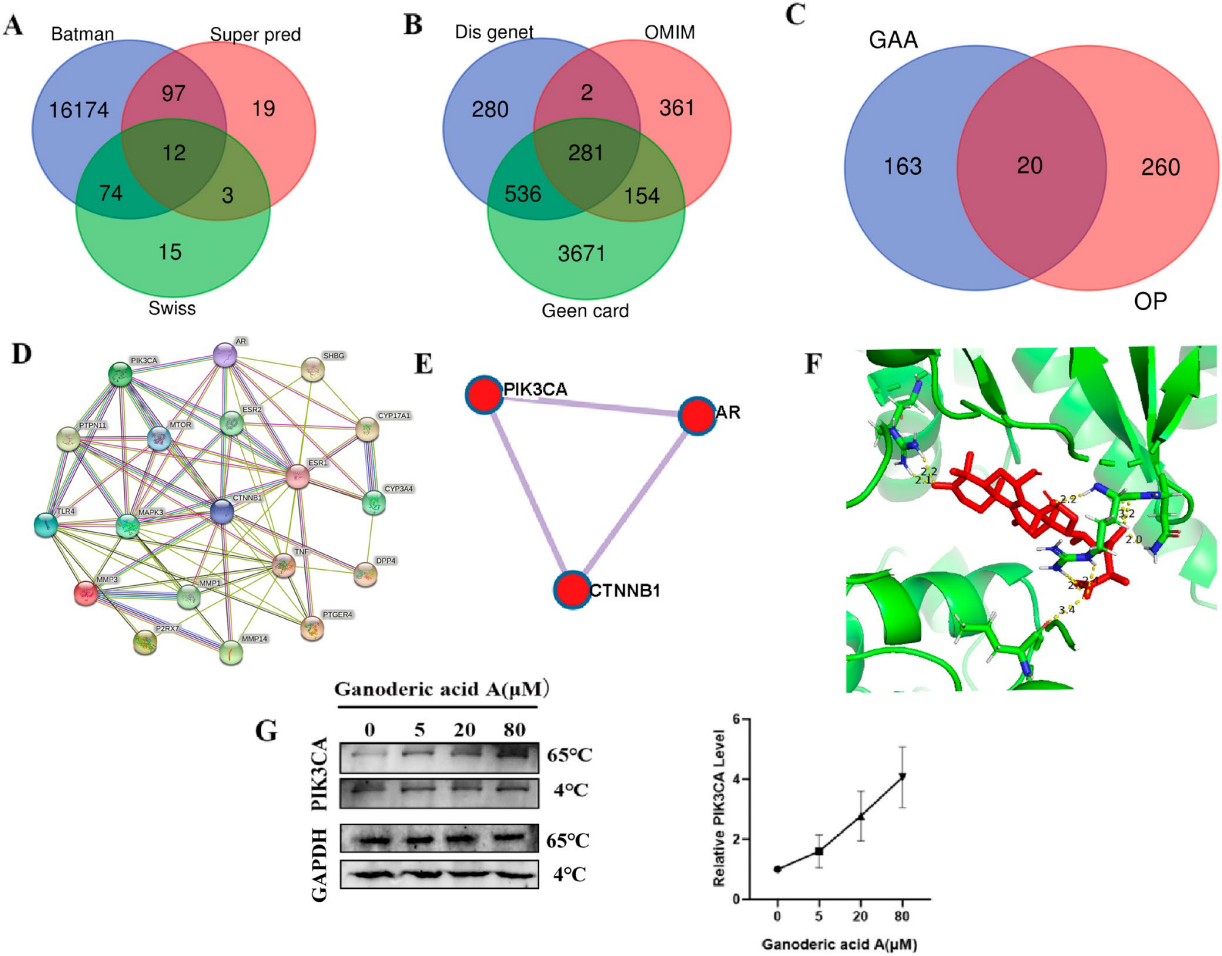
The workflow for screening target genes using the network pharmacology and computational bioinformatics analysis approach. (A) Identification of GA‐A‐related targets. (B) Identification of OP‐related targets. (C) Obtaining 20 GA‐A–OP‐related targets. (D) PPI network construction and analysis. (E) Analysis of the 3 center targets in the PPI subnetwork. (F) Molecular docking of the PIK3CA center target in the PPI subnetwork. (G) Dose–Response CETSA Experiment on the PIK3CA protein.

Then the mechanisms of GA‐A suppressing bone loss were investigated. Firstly, bioinformatics was used to analyze the targets of GA‐A protecting against osteoporosis. Through Batman, SuperPred, and Swiss websites, the acting targets of Ganoderic acid A were obtained respectively, and the intersection was selected to obtain the Venn diagram (Figure 2A). The targets of osteoporosis were obtained through Disgenet, OMIM, and Gene card websites, respectively, and the intersection was selected to obtain the Venn diagram (Figure 2B). The Venn intersection results of GA‐A and osteoporosis were performed to obtain the common targets between Ganoderic acid A and osteoporosis (Figure 2C). The String database was used to construct a protein–protein interaction (PPI) of the common targets and 3 central targets were identified. According to the relevant literature, the effects of AR and CTNNB1 have already been studied well in the related studies (Huang et al. 2023; Chen et al. 2003; Liu et al. 2019), while the effects of PIK3CA still remain unclear in osteoporosis (Figure 2D,E). Moreover, the results of molecular docking showed that the binding energy of PIK3CA to GA‐A was −7.12 kcal/mol, foreshadowing that this protein is likely to be the target of Ganoderic acid A against OP (Figure 2F). Subsequently, we performed the Dose–Response CETSA experiment on the PIK3CA protein, and the results showed that GA‐A could make the protein more stable, which proved that PIK3CA could bind with GA‐A (Figure 2G). Therefore, PIK3CA was selected for the subsequent mechanistic study for GA‐A against osteoporosis.

### 3.3 Ganoderic Acid A Modulates the PIK3CA/p‐Akt/TWIST1 Signaling Pathway in the OVX Mice

**FIGURE 3:**
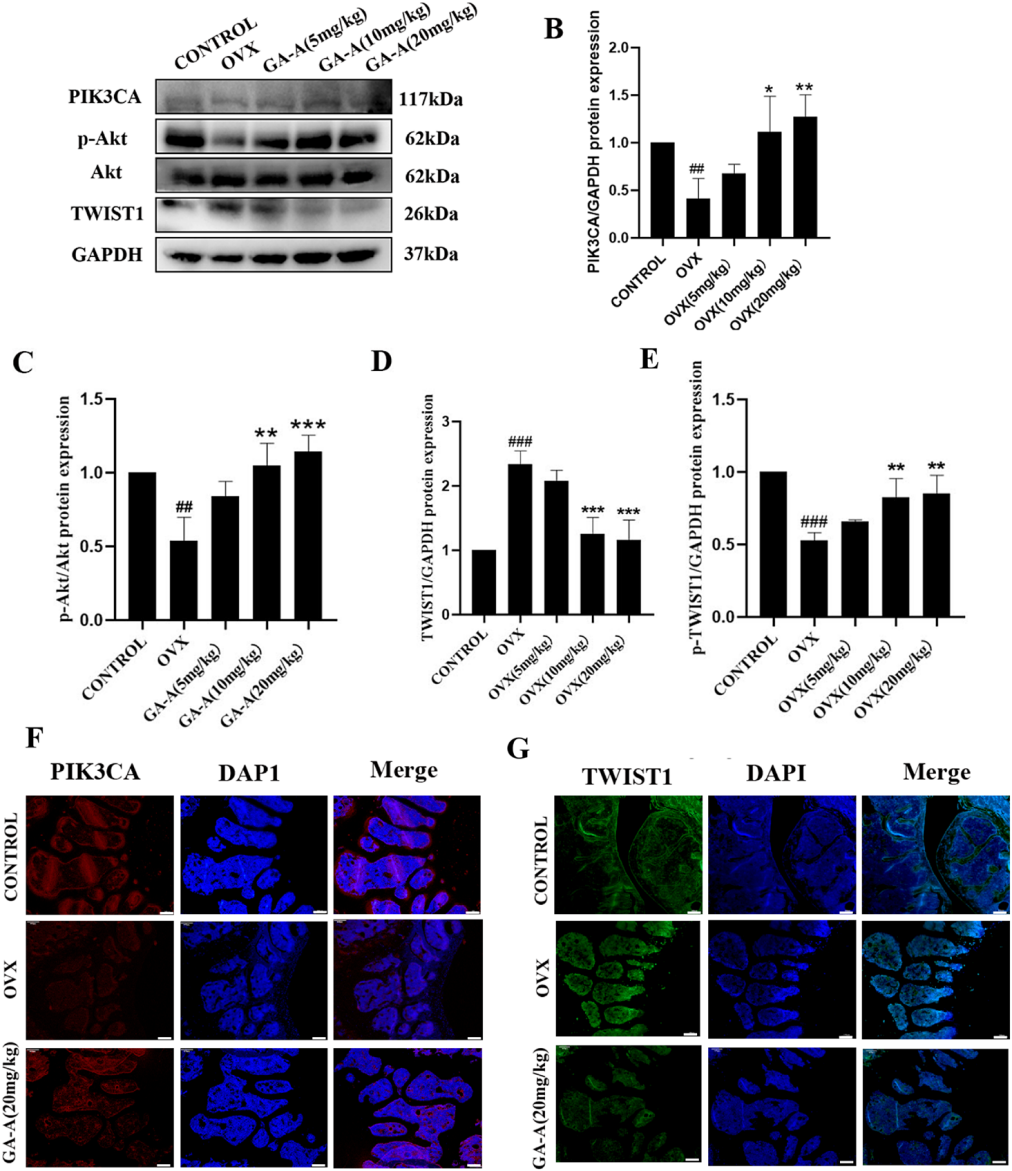
Validation of GA‐A's therapeutic target for OP. (A, B) Putative target PIK3CA validation by Western blot analysis. (A, C, D, E) Detection of p‐Akt, TWIST1, and p‐TWIST1 indicators by western blot. (F, G) Fluorescence intensity visualization of PIK3CA and TWIST1 production by fluorescent microscope *n* = 6, # *p* < 0.05, ##*p* < 0.01, and ###*p* < 0.001 compared with the control group. *p < 0.05 and **p < 0.01 compared with the OVX group (scare bar = 100 μm).

The feasibility of the above screening targets was first verified by Western blot in vivo. The results showed that the protein expression of PIK3CA was significantly downregulated in the OVX group. However, it was up‐regulated significantly after the administration of gradient Ganoderic acid A (Figure 3A,B), which suggested that PIK3CA was possibly the acting target of GA‐A and PIK3CA was positively related to osteoblastic differentiation. Next, the Western blot results verified the decreased ratio of p‐Akt/Akt and the increased protein expression of TWIST1 in the OVX mice, which could be reversed by GA‐A treatment (Figure 3C–E). Then the immunofluorescence experiments were used to confirm the result. The results demonstrated consistent results with the protein expressions in Western blot analysis (Figure 3F,G). These results further supported the feasibility of GA‐A acting on the PIK3CA/p‐Akt/TWIST1 signaling pathway to prevent bone loss induced by OVX.

### 3.4 GA‐A Promotes Osteogenic Differentiation in the MC3T3‐E1 Cells and Primary Osteoblasts

**FIGURE 4:**
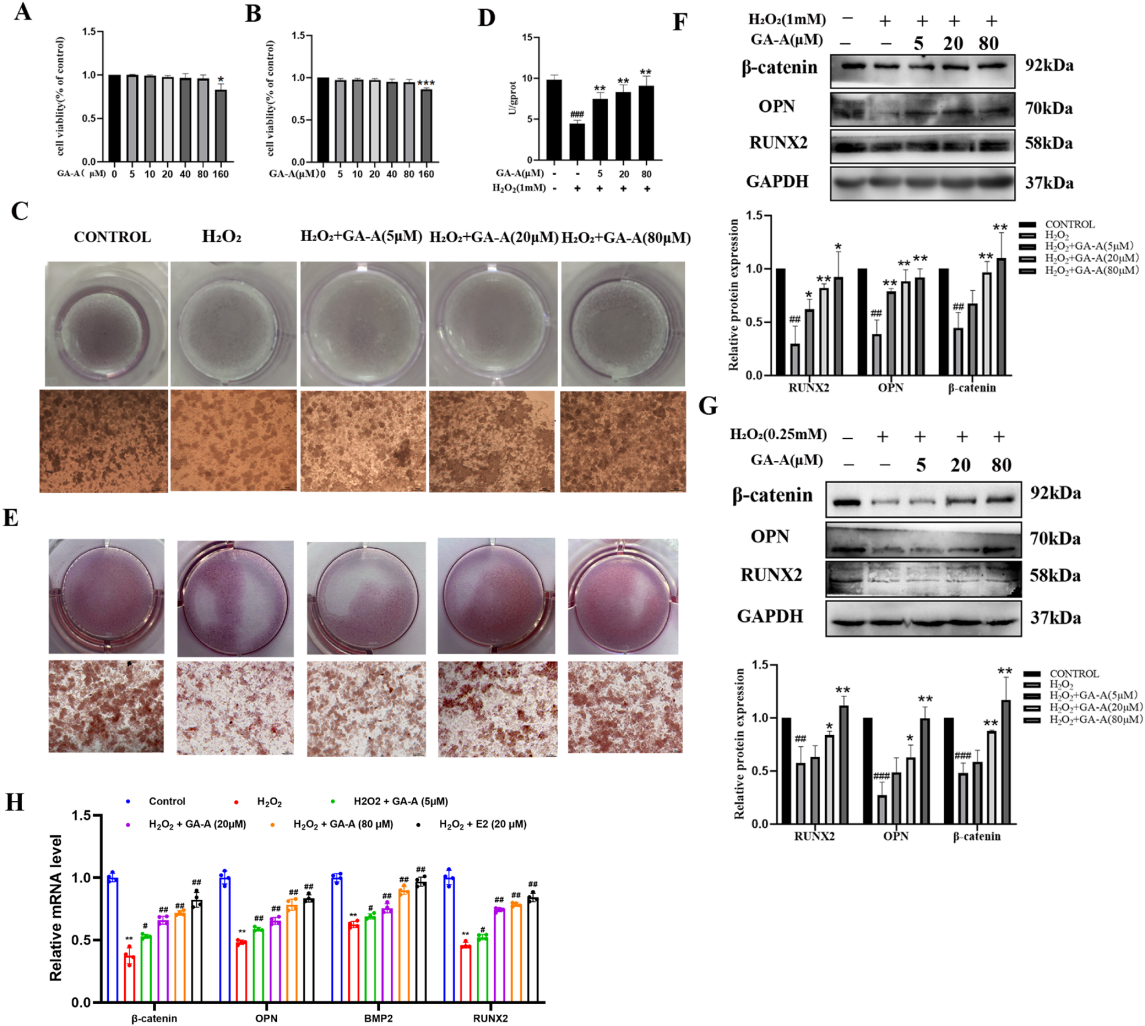
GA‐A suppressed H_2_O_2_‐induced inhibition of osteogenesis differentiation and mineralization in MC3T3‐E1s and primary osteoblasts. (A, B) CCK‐8 analysis. (C) ALP staining and (D) ALP activity detection. (E) ARS staining of MC3T3‐E1s. (F, G) Protein expression of osteogenesis‐related markers RUNX2, OPN, and β‐catenin in MC3T3‐E1s and primary osteoblasts. *n* = 6, #*p* < 0.05, ##*p* < 0.01 and ###*p* < 0.001 compared with the control group. *p < 0.05 and **p < 0.01 compared with the H_2_O_2_ group. (H) qPCR assay of RUNX2, OPN, BMP2, and β‐catenin after treated with E2 or GA‐A in MC3T3‐E1 (*p < 0.05 and **p < 0.01 compared with the control group. #*p* < 0.05 and ##*p* < 0.01 compared with the H_2_O_2_ group).

MC cell line and primary osteoblasts were used to verify the effects of GA‐A promoting osteogenic formation in vitro, and H_2_O_2_ was used to induce oxidative stress injury in the cells (Sies 2017). According to the results of the cck8 assay, the concentration of H_2_O_2_ used in the MC cells was 1 mM, and in POB was 250 μM. Also, 5, 20, and 80 μM of GA‐A were used for the following study (Figure 4A,B).

In MC cells, low, medium, and high doses of GA‐A were given for 24 h, followed by 4 h of H_2_O_2_ exposure. Then the cells were induced for 14 days to detect alkaline phosphatase (ALP) activity by ALP staining and an ALP detecting kit. Alizarin red (ARS) staining was performed 21 days after induction. The results of the ALP assay showed that compared with the control group, the number of dark staining nodules of ALP was significantly reduced and the ALP activity was significantly decreased in the H_2_O_2_‐ induced group; however, GA‐A could increase the number of dark staining nodules and ALP activity in a dose‐dependent manner. Alizarin red results also showed that the number of mineralized nodules in the model group was significantly reduced, and the color became lighter. Remarkably, in the GA‐A treatment group, the number of mineralized nodules was obviously increased and the color was deepened in a dose‐dependent manner (Figure 4C–E). Consistent with the in vivo results, the protein expressions of osteogenic related markers RUNX2, OPN, and β‐catenin in both cell lines were decreased after exposure to H_2_O_2_ detected by Western blot analysis, whereas GA‐A acted in an opposite way (Figure 4 F, G). What is more, when compared with estrogen (E2), an approved medicine used for the treatment of postmenopausal OP, a high dose of GA‐A (80 μM) exhibited almost the same effects on osteogenic related markers (Figure 4H). These results implied that GA‐A could promote osteogenic formation in H_2_O_2_‐induced MC3T3‐E1 cells and primary osteoblasts.

### 3.5 Effects of GA‐A on the PIK3CA/ Akt/TWIST1 Signaling Pathway In Vitro

**FIGURE 5:**
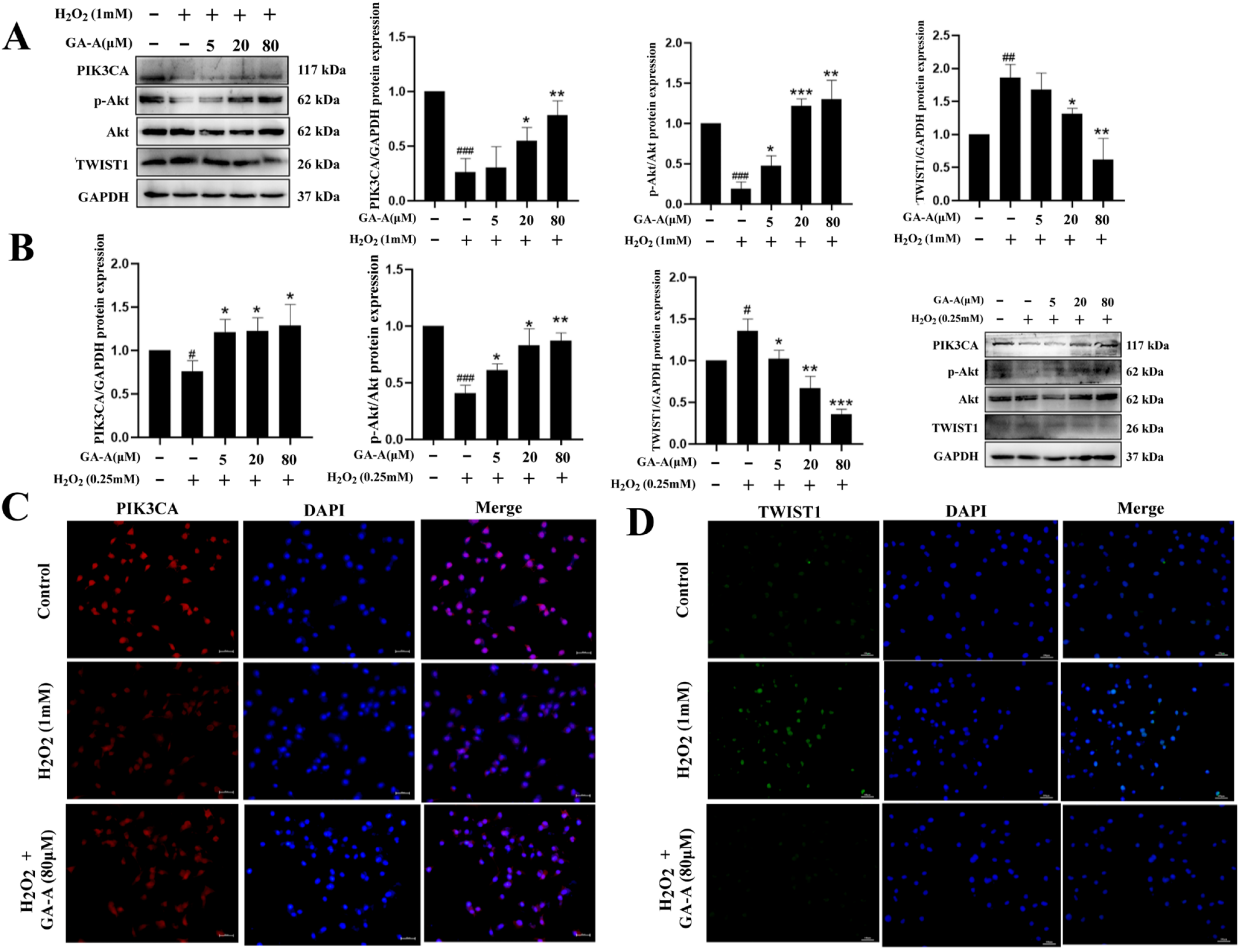
Validation of GA‐A's therapeutic target for OP in MC3T3‐E1s and primary osteoblasts. (A) Putative target PIK3CA, p‐Akt, TWIST1, and p‐TWIST1 validation by Western blot in MC3T3‐E1s. (B) Putative target PIK3CA, p‐Akt, TWIST1, and P‐TWIST1 validation by Western blot in primary osteoblasts. (C, D) Fluorescence intensity visualization of PIK3CA and TWIST1 production by fluorescent microscope in MC3T3‐E1 cells. *n* = 6, # *p* < 0.05, ##*p* < 0.01 and ###*p* < 0.001 compared with the control group. *p < 0.05, **p < 0.01 and ***p < 0.001 compared with the H_2_O_2_ group.

The expression of the target proteins in MC cells and POB was also detected by Western blotting. The results showed that the changes in the target proteins were consistent in both cell lines. Compared with the control group, PIK3CA and p‐Akt protein expressions in the model group decreased, and they were increased in a dose‐dependent manner after GA‐A treatment; However, the protein expression of TWIST1 was contrary to that of PIK3CA (Figure 5A,B). In order to further verify the expression changes of the above signal proteins, the fluorescence intensity of PIK3CA and TWIST1 was then identified by immunofluorescence staining. The results showed that the fluorescence intensity of both proteins was consistent with the in vivo immunofluorescence results, with a decrease in the fluorescence intensity of PIK3CA and an increase in the fluorescence intensity of TWIST1 in the H_2_O_2_‐induced group, which was reversed by GA‐A administration (Figure 5C,D).

### 3.6 PIK3CA Promotes Osteogenic Differentiation via Increasing Akt Phosphorylation to Down‐Regulate TWIST1 Expression in MC3T3‐E1 Cells

**FIGURE 6:**
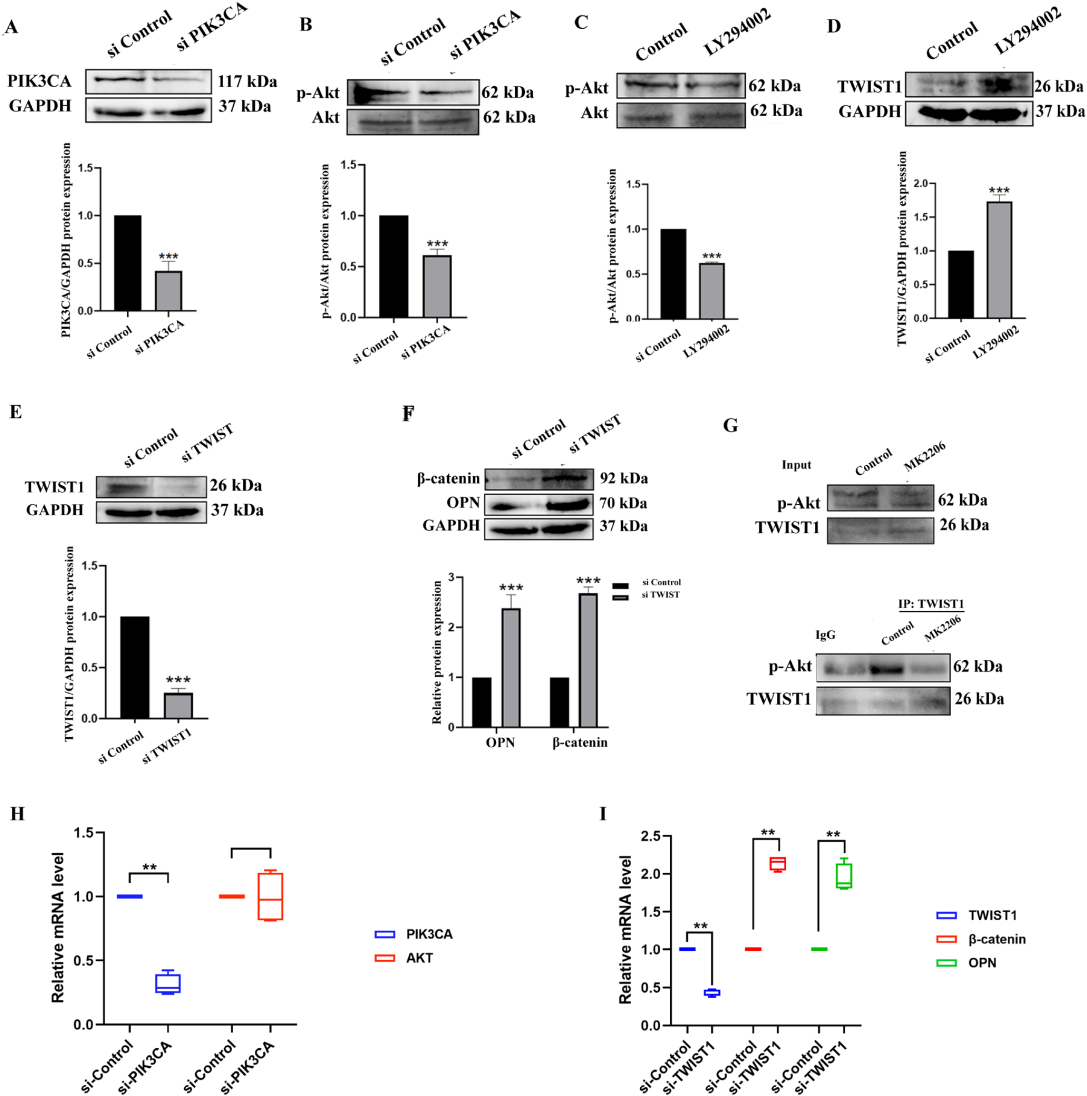
The mechanism of PIK3CA's inhibiting for OP. (A, B) Knockdown of PIK3CA by siRNA and detection of PIK3CA and p‐Akt indicators by Western blot. *n* = 6, ***p < 0.001 compared with the si‐Control group. (C, D) PI3K/Akt inhibitor (LY294002) inhibits p‐Akt and detection of p‐Akt, TWIST1, and p‐TWIST1 indicators by Western blot. *n* = 6, ***p < 0.001 compared with the control group. (E, F) Knockdown of TWIST1 by siRNA and detection of osteogenesis‐related indicators by Western blot. *n* = 6, ***p < 0.001 compared with the si‐Control group. (G) Detection of the protein interaction of p‐Akt with TWIST1 by Co‐IP western blot. (H) qPCR assay of PIK3CA and Akt after PIK3CA knocking down (*n* = 4, **p < 0.001 compared with the control group). (I) qPCR assay of β‐catenin and OPN after Twist1 knocking down (*n* = 4, **p < 0.001 compared with the control group).

To investigate the specific mechanism of GA‐A activating PIK3CA signaling protein on bone formation, the si‐PIK3CA silencing sequence was transfected into the MC cells. The silencing efficiency of PIK3CA in the cells was detected by Western blot and qPCR, and the result showed that the silencing efficiency was about 50% (Figure 6A). After the knockdown of PIK3CA, the expression of p‐Akt was also significantly decreased, indicating that PIK3CA could activate the PI3K/Akt signaling pathway to upregulate the protein expression of p‐Akt (Figure 6B,H).

Next, to verify the regulating effect of p‐Akt on TWIST1, p‐Akt expression was inhibited with the PI3K/Akt inhibitor (LY294002) (Figure 6C). According to existing research, phosphorylation of TWIST1 by Akt is required for β‐TrCP‐mediated TWIST1 ubiquitination and degradation (Li et al. 2016). Therefore, after the p‐Akt protein was inhibited, we examined p‐TWIST1 protein and TWIST1 protein and found that p‐TWIST1 level was significantly decreased, while TWIST1 level was significantly increased, suggesting that p‐Akt regulates TWIST1 by phosphorylating it for degradation (Figure 6D).

Finally, to verify the effects of TWIST1 on bone formation, the si‐TWIST1 silencing sequence was transfected into the cells by transfection technology, and the silencing efficiency of TWIST1 in the cells was detected by Western blot and qPCR. The result showed that the silencing efficiency was about 80% (Figure 6E). Expectedly, after the knockdown of TWIST1, the expression of OPN and β‐catenin in the si‐TWIST1 group was significantly higher than that in the control group (Figure 6F,R), confirming that TWIST1 negatively regulates osteogenic differentiation.

Some studies have found that the expression of the transcription factor TWIST1 is correlated with p‐Akt (Yuan et al. 2014; Inoue et al. 2017). However, the specific relationship between the two factors has not been proven. To verify the relationship between p‐Akt and TWIST, the Co‐IP experiment was used to verify the interaction of the two factors. As shown in the results, p‐Akt could act on TWIST1 to affect its expression, which was consistent with the experimental results of the input group (Figure 6G).

Therefore, these results suggested that Ganoderic acid A could promote osteoblastic bone formation and protect against osteoporosis through activating PIK3CA and subsequently activating the PI3K/Akt signaling pathway to down‐regulate TWIST1 expression.

## 4 Discussion

Osteoporosis is a disease positively related to bone loss and deterioration of bone microstructure, which seriously endangers human health. Prevention and treatment of osteoporosis have gradually become a public concern. In recent years, more and more natural extracts of traditional Chinese medicine have become a hot topic of anti‐osteoporosis. Epimedium, *Lycium barbarum* , *Pueraria lobata* , Angelica sinensis, *Eucommia ulmoides* , and other active ingredients have been reported to be safe and effective in the prevention and control of osteoporosis (Indran et al. 2016; Li et al. 2022; Lim and Kim 2014; He et al. 2019). As the king of traditional Chinese medicine, Ganoderma lucidum can treat various diseases, and it is reported to be able to inhibit bone loss in the glucocorticoid‐induced osteoporosis model (Yang et al. 2020). However, the molecular mechanism of Ganoderma lucidum against osteoporosis still remains unknown. Therefore, the present study was to investigate the effects and underlying mechanisms of GA‐A, one of the main active ingredients of Ganoderma lucidum, inhibiting osteoporosis.

The model established by ovariectomy is one of the common osteoporosis models, which can simulate the formation of primary osteoporosis. Therefore, this model was used in our study to verify the preventive and therapeutic effects of Ganoderic acid A on osteoporosis. H&E staining results demonstrated that the bone tissue structure of the OVX model group was fractured, with a large number of fat cavities and a significant reduction in bone density. Micro‐CT imaging results also showed that the bone microstructure was severely damaged, and the bone mineral density of the femur was significantly reduced in the model group. However, after Ganoderic acid A treatment, these phenomena were significantly antagonized in a dose‐dependent manner. ALP in the serum is mainly derived from the liver and bone, and high levels of ALP can lead to increased osteocyte activity and are associated with decreased bone mass (Llorente‐Pelayo et al. 2020). In the present study, the serum ALP level was significantly increased in the OVX group, suggesting the decreased bone mass, and consistent with the above results, serum ALP levels were obviously suppressed in a dose‐dependent manner after Ganoderic acid A treatment.

RUNX2, OPN, and β‐catenin are osteogenesis‐related proteins that are related to bone formation. In this experiment, these protein expressions in the model group were significantly decreased, suggesting that the osteoporosis model was successfully established and bone formation was significantly reduced. After Ganoderic acid A administration, the protein expression levels of osteogenic‐related markers significantly increased, indicating that Ganoderic acid A could alleviate OVX‐induced osteoporosis.

In the process of studying the treatment of osteoporosis with traditional Chinese medicine and active ingredients, the targets of drugs can be screened by bioinformatics methods, and then the targets can be verified following subsequent research (Wang et al. 2023). Therefore, in this study, the protective effects of GA‐A, the main active ingredient of Ganoderma lucidum, on osteoporosis and the underlying mechanisms were investigated based on network pharmacology.

In the screening of pharmacological targets in the GA‐A network, PIK3CA was selected as the target protein. Previous studies have found that in glucocorticoid‐induced osteoporosis, icariin can activate PIK3CA, thereby inhibiting osteoporosis (Hu et al. 2017). Therefore, it was reasonable to speculate that GA‐A could also bind to PIK3CA and activate the protein, which was confirmed in the subsequent experiments. Both molecular docking and Western blotting experiments showed that the anti‐osteoporosis effects of GA‐A were generated through the upregulation of PIK3CA protein expression. PIK3CA might be a target for the prevention and treatment of osteoporosis.

PIK3CA was found to act as a catalytic subunit of PI3K and can activate PI3K, an important member of the PI3K/Akt signaling pathway, to activate the Akt protein into a phosphorylated state. The PI3K/Akt signaling pathway plays a key role in many physiological processes, including the regulation of bone formation (Dou et al. 2021; Liu et al. 2021; Zhao et al. 2020). Therefore, the expression of the Akt protein in this signaling pathway was detected, and the results showed that the phosphorylation level of the Akt protein in the OVX group was significantly lower than that in the sham group and was gradually improved after the administration of GA‐A, which proved that GA‐A had a positive correlation with the expression of this signaling protein.

PI3K/Akt pathway can also regulate the expression of many downstream proteins and affect the occurrence and development of a variety of diseases. After reviewing a large number of literatures, the transcription factor TWIST1 was found to be associated with p‐Akt expression (Yuan et al. 2014; Inoue et al. 2017). TWIST1, a highly conserved member of the TWIST subfamily of basic helix–loop–helix transcription factors, regulates bone development and can inhibit osteoblast activity in many models (Zhu et al. 2012; Croset et al. 2014). Therefore, the expression of TWIST1 in the ovariectomy‐induced model was verified, which was consistent with the data reported that TWIST1 is an osteogenic negative related protein (Yang et al. 2017), and the protein expression of TWIST1 could be reversed after GA‐A treatment. The effect of GA‐A on TWIST1 protein expression was also consistent with the above results in vitro.

Next, in vitro experiments were performed to confirm the specific mechanism of action. The results showed that the protein expression of p‐Akt was significantly inhibited after si‐PIKCA treatment. We subsequently found that TWIST1 expression was significantly increased after treatment with the p‐Akt inhibitor LY294002, and the protein levels of bone formation‐related markers were significantly increased in osteoblasts after knockdown of TWIST1. These results indicate that Ganoderma acid A inhibits TWIST1 expression by activating PIK3CA, thereby activating the PI3K/Akt signaling pathway, thereby inhibiting bone loss and promoting bone formation.

This experimental study has the following limitations. PIK3CA‐mediated activation of the PI3K/Akt signaling pathway not only plays a key role in the process of osteogenesis but also regulates the process of bone mass loss by osteoclasts. Therefore, the pharmacological effects of GA‐A on osteoclasts can be further explored in future studies. Whether PIK3CA, as a target of GA‐A against osteoporosis, can also be involved in the inhibition of osteoclast formation needs further investigation.

## 5 Conclusion

This study found that Ganoderic acid A could inhibit the activity of TWIST1 in the OVX mice and H_2_O_2_‐induced osteoblasts by upregulating the PIK3CA target protein and activating the PI3K/Akt signaling pathway, thereby inhibiting bone loss and promoting bone formation. Ganoderic acid A provides potential drug treatment for the prevention and treatment of postmenopausal osteoporosis.

## Author Contributions

Jianyu Zhao, Ying Fan, and Changyuan Wang performed the experiment, collected, and interpreted the data; Jianyu Zhao, Hao Li, Sihang Fan, and Qingchen Li provided technical support and helped perform the analysis with constructive discussions; Mozhen Liu and Huijun Sun designed the study, obtained funding, supervised the study, drafted, and critically revised the paper; All data were generated in‐house, and no paper mill was used. All authors have read and approved the final manuscript.

## Ethics Statement

All animal experiments were conducted according to the Guide for the Care and Use of Laboratory Animals and approved by the Animal Research Ethics Committee of Dalian Medical University (Dalian, China; ethical No. AEE21084).

## Conflicts of Interest

The authors declare no conflicts of interest.

## Data Availability

The data that support the findings of this study are available on request from the corresponding author.
